# Label Free Detection of CD4+ and CD8+ T Cells Using the Optofluidic Ring Resonator

**DOI:** 10.3390/s100605798

**Published:** 2010-06-08

**Authors:** John T. Gohring, Xudong Fan

**Affiliations:** 1 Department of Biological Engineering, 240D Bond Life Sciences Center, 1201 East Rollins Street, University of Missouri, Columbia, MO 65211, USA; E-Mail: jtgdg5@mizzou.edu; 2 Department of Biomedical Engineering, 2158 Lurie Biomedical Engineering Building, 1101 Beal Ave., University of Michigan, Ann Arbor, MI 48109, USA

**Keywords:** HIV, CD4, CD8, ring resonator, optical biosensor, label free

## Abstract

We have demonstrated label free detection of CD4+ and CD8+ T-Lymphocyte whole cells and CD4+ T-Lymphocyte cell lysis using the optofluidic ring resonator (OFRR) sensor. The OFRR sensing platform incorporates microfluidics and photonics in a setup that utilizes small sample volume and achieves a fast detection time. In this work, white blood cells were isolated from healthy blood and the concentrations were adjusted to match T-Lymphocyte levels of individuals infected with HIV. Detection was accomplished by immobilizing CD4 and CD8 antibodies on the inner surface of the OFRR. Sensing results show excellent detection of CD4+ and CD8+ T-Lymphocyte cells at medically significant concentrations with a detection time of approximately 30 minutes. This work will lead to a rapid and low-cost sensing device that can provide a CD4 and CD8 count as a measure of HIV progression.

## 1. Introduction

The ability to detect and quantify HIV infection is essential to physicians seeking to maximize the impact of treatment and accuracy of prognosis. One way to achieve this is to look at the counts of CD4+ T-Lymphocyte cells as biological indictors of the progression of HIV [1]. Normal CD4+ counts in healthy individuals are around 1,200 CD4+ cells per μL [2–4]. Infected individuals usually begin Highly Active Anti-Retroviral Treatment (HAART) when CD4+ concentration levels drop into the range of 200 to 300 CD4+ cells per μL and are diagnosed as having AIDS when these counts fall below 200 CD4+ cells per μL. HAART can be initiated at higher CD4+ counts (300–800 CD4+ cells per μL) if it is deemed necessary by the physician [3–7].

Another test that may be used in conjunction with a CD4+ count is the CD8+ T-Lymphocyte count, which might be able to assess the risk of rapid CD4+ cell depletion and susceptibility to developing AIDS [8–10]. CD8+ levels typically rise as HIV infection progresses [9,11]. In a healthy individual there are normally 1–2 CD4+ cells to every 1 CD8+ cell. This number can be expressed as a ratio of CD4+/CD8+. As this ratio decreases, it may be a sign that the immune system is becoming weaker and more prone to opportunistic infection [9,11]. It has been suggested that after CD4+ counts fall below the AIDS threshold CD8+ cell levels will begin to drop from an elevated level [9,12]. Monitoring these falling CD8+ cell levels late in HIV infection can give doctors a measure of virus severity and tools for a more accurate prognosis [9,11,12].

Presently, CD4+ and CD8+ counts can be accurately established in a clinical setting using a flow cytometer [7,13]. These machines work by labeling CD4+ or CD8+ receptors with fluorescent antibodies and counting the resulting fluorescent signal. While flow cytometers accomplish these counts with great accuracy and high throughput, they have intrinsic limitations such as high equipment cost, the need for specialized personnel, and high maintenance requirements [13]. These setbacks make such equipment unusable in under-developed countries where HIV has reached pandemic levels. In these countries there is a great need for devices that can detect CD4+ and CD8+ counts as a measure of HIV progression in a rapid, easy to use, and low cost setup.

In the past few years there have been many studies related to CD4+ cell detection focusing on developing low cost CD4+ detection alternatives that perform well compared to established detection technologies [1]. One method that is being heavily researched to improve the ease and size requirements of detection is combining quantum dot (QD) fluorescent technology with lab on a chip based bio-detection platforms [14]. Properties such as photostability and ease of capturing target receptors make QDs optimal for imaging target molecules in a small setup [14]. Another method recently studied for CD4 cell detection uses cell affinity chromatography in a microfluidic chip based platform [3]. This technology relies on fluorescent labeling of cells captured by a differential shear flow in a microfluidic channel specifically calibrated for CD4 isolation [3]. Expanding upon lab-on-a-chip based platforms, devices utilizing metal oxide semiconducting field effect transistors (MOSFETs) to obtain fast CD4+ cell counts and fluorescent technologies to verify cell concentrations have also been demonstrated [15]. While these methods show promise, the technologies rely heavily on fluorescent tagging and imaging to achieve detection. These labeling procedures add to the complexity of the detection mechanism by requiring expensive QDs, fluorescent labels, and imaging equipment [3].

**Figure 1.**
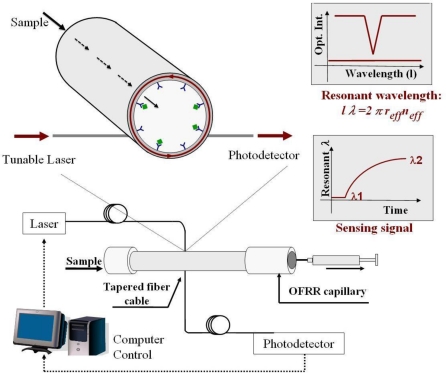
Schematic of the OFRR, sensing signal and experimental setup for cell detection.

In this work we have developed an optofluidic ring resonator (OFRR) sensor for label free detection of CD4+ and CD8+ T-Lymphocyte whole cells and CD4+ T-Lymphocyte cell lysis that addresses some of the weaknesses described above. The OFRR is an optical sensing platform that integrates mircofluidics with an optical ring resonator technology [16]. Shown in Figure 1, a tapered fiber cable couples light into the capillary wall where it is confined in circular resonances known as whispering gallery modes (WGMs). When the input light matches the resonant condition of the WGM, a dip in light intensity is observed at the end of the tapered fiber cable. The spectral position of this mode can be used to track the sensing signal in real time. While the WGM is resonating, an evanescent field will extend about 100 nm from the inner capillary interface. Because of this, the glass wall of the OFRR needs to be thin enough (<4 μm) to allow an evanescent field to extend into the microfluidic channel and interact with analyte.

OFRR devices achieve label free sensing by detecting the refractive index (RI) changes in the microfluidic channel. This change in RI can be caused by a change in the overall fluid in the core or a buildup of biomolecules on the surface of the channel. The relationship between the spectral shift of the WGM and the RI of the microfluidic core is shown mathematically by [17–19]:

(1) $$l\lambda =2\pi r_{\text{eff}}\;n_{\text{eff}}$$

where *n*_eff_ is the effective RI of the solution sensed by the WGMs, *r*_eff_ is the radius of the capillary, *l* is an integer representing the angular momentum of the WGM, and λ is the resonant wavelength of the WGM. As biomolecules bind to the inner surface of the OFRR’s microfluidic channel, they interact with the evanescent field. The accumulation of a biomolecule layer changes the *n*_eff,_ leading to measurable change in λ due to a WGM resonant wavelength shift. The OFRR has been used to detect protein [19], DNA [20], viral particle [18], and pesticide concentrations [21] with a RI detection limit on the order of 10^−7^ refractive index units (RIU) and mass detection on the order of pg mm^−2^ [16,18,20–22].

In addition to excellent performance, the OFRR has several essential properties that make it an easy to use and low cost alternative for CD4+ and CD8+ detection. The use of mature telecom optical equipment contributes to the low cost of the overall setup and availability of setup components. Having the ring resonator incorporated with a microfluidic channel that is only a hundred micrometers in diameter provides a small device size that uses a very small sample to achieve detection and a short assay time. Because of these factors and the label free characteristics of the system, the OFRR could potentially be used in third world countries where resources are limited. The OFRR could be used as a simple point-of-care device to monitor HIV progression and give doctors operating in these countries a tool for prognosis that exhibits CD4+ and CD8+ detection with results comparable to more expensive and resource heavy equipment.

In this paper we describe the use of the OFRR for rapid identification and quantification of CD4+ and CD8+ T-Lymphocyte whole cells and CD4+ T-Lymphocyte cell lysis. The results show that the OFRR achieves accurate CD4+ and CD8+ counts at levels associated with HIV infection and the medically accepted levels to begin HAART and the AIDS threshold.

## 2. Results and Discussion

### 2.1. Detection of CD4+ T-Lymphocytes

**Figure 2.**
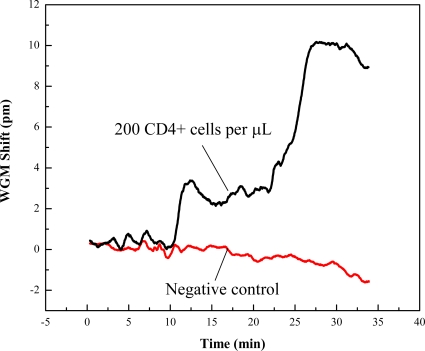
Sensorgram trace of a positive control sample containing approximately 200 CD4+ cells per μL. Negative control consists of diluted serum and red blood cells.

**Figure 3.**
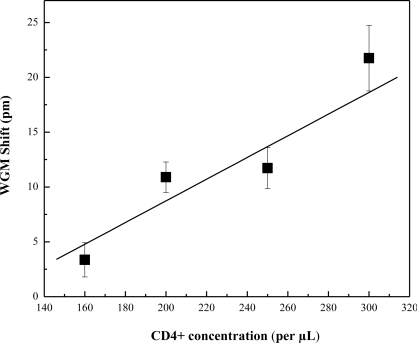
Spectral shift recorded for various concentrations of CD4+ T-Lymphocytes in PBS buffer. Error bars are based on standard deviation obtained from 10 measurements.

The OFRR was engaged for the real time detection of CD4+ T-Lymphocytes. The interior surface was functionalized using the procedure described in the experimental section. A detection baseline was created by running PBS buffer through the OFRR. Lymphocyte cell concentrations were verified using a hemacytometer and microscope. A level of cell viability was assessed by staining dead cells with Trypan Blue. It was found that in each experiment a high level of cell viability was present. After concentrations of lymphocytes were measured, they were diluted to standard concentrations in medically significant ranges pertaining to individuals infected with HIV. The concentrations chosen were approximately 160, 200, 250, and 300 cells per μL. The resulting WGM spectral shift was recorded for each concentration and range was established based on multiple tests. Figure 2 shows one of the sensorgrams when CD4+ cells bind to the OFRR inner surface. During the test, sample flowing through the OFRR was held constant by the syringe pump at 1 μL/min. The whole test was terminated after approximately 30 minutes. The sensorgram exhibits much larger fluctuations in the sensing signal (*i.e*., the WGM spectral shift) than normally seen in detection of DNA or protein. This is caused by the non-uniform nature of cell binding processes, as compared to the binding of biomolecules. In order to establish the specificity of CD4+ T-Lymphocyte binding to the OFRR sensing region, we ran a negative control consisting of diluted serum and red blood cells in PBS buffer. Figure 3 shows the resulting spectral shift ranges at varying concentrations. It is seen that the sensing signal is linearly dependent upon the cell concentration, although error bars are relatively large due once again to the nature of the cell binding.

### 2.2. Detection of CD8+ T-Lymphocyte

**Figure 4.**
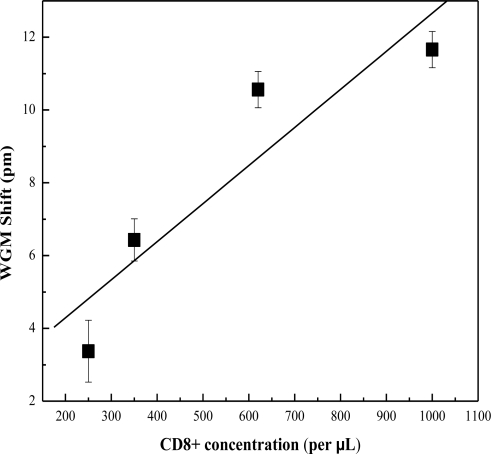
Spectral shift recorded for various concentrations of CD8+ T-Lymphocytes. Error bars are based on standard deviation obtained from 10 measurements.

Detection of CD8+ T-Lymphocytes was determined by the OFRR using a procedure similar to CD4+ detection. The OFRR was functionalized using the procedure outlined in the experimental section of this paper. Concentrations of lymphocytes were established using hemacytometer and cell viability was determined by staining dead cells with Trypan Blue. Cell viability was high during the CD8+ detection tests. CD8+ concentrations were then prepared replicating probable CD8+ concentrations of HIV infected individuals. The sample was pulled through the OFRR with a syringe pump at a rate of 1 μL/min. Multiple tests were completed to establish a range of sensing signal at approximate CD8+ concentrations. Figure 4 shows the results of CD8+ detection at various concentrations.

### 2.3. CD4 Lysis Measurements

**Figure 5.**
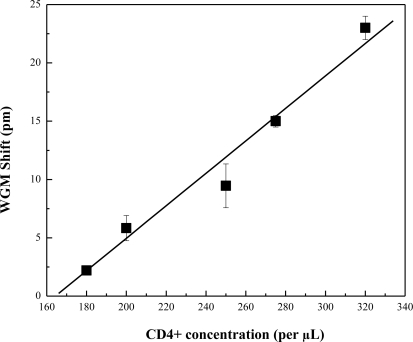
Spectral shift recorded for concentrations of CD4+ T-Lymphocyte cell lysis. Error bars are based on standard deviation obtained from 5 measurements.

The OFRR was used in the detection of CD4+ T-Lymphocyte cells that were lysed. Lysing the whole cells allows for the detection of individual cell surface receptors and will allow for more uniform arrival of sample to the sensing area. This could reduce error bars and allow for easier detection at the AIDS concentration threshold with smaller fluctuations. The OFRR was functionalized in the same way that was used for whole cell CD4+ T-Lymphocyte detection. Concentrations of CD4+ cells were established with a hemacytometer and were then lysed using a lymphocyte lysis buffer. Various concentrations of CD4+ cell lysis based on the CD4+ whole cell count were then passed through the OFRR. The concentrations tested were 180, 200, 250, 280, and 320 cells per μL. Samples were pulled through the OFRR with a syringe pump at a rate of 1 μL/ min. Multiple tests were performed to establish error bars based on the standard deviation. The results of this experiment are shown in Figure 5. Results show that we achieve the reduction of error bars calculated from standard deviation at the same concentrations used in CD4+ whole cell detection.

## 3. Experimental

### 3.1. Materials

Ninety-eight percents Ethanol, 47% hydrofluoric acid (HF), 3-aminopropyltrimethoxysilane (3-APS), Trypan Blue, and recombinant protein G were purchased from Sigma-Aldrich (St. Louis, MO). Dimethyl pimelimidate dihydrochloride (DMP) cross linker was purchased from Pierce Biotechnology (Rockford, IL). Lysis buffer consisting of Tris-HCL, Triton X-100, MgCl_2_, and sucrose used materials obtained from Sigma Aldrich. CD4 mouse monoclonal IgG antibodies were purchased from Santa Cruz Biotechnology (Santa Cruz, CA) and CD8 mouse monoclonal antibodies were purchased from AbCam (Cambridge, MA). White blood cells were isolated from whole blood drawn at Ellis Fischel Cancer Center (Columbia, MO). Concentrations of lymphocytes were verified using a hemacytometer in lab purchased from Sigma-Aldrich. Silica glass tubes of 1.2 mm outer diameter and 0.85 mm inner diameter were purchased from Sutter Instruments (Novato, CA).

### 3.2. Experimental Setup

The OFRR was fabricated by pulling a quartz tube under intense heat generated by two CO_2_ lasers creating a capillary with a 150 μm outside diameter. In order to increase the sensitivity of the OFRR to achieve biomolecule detection, the wall thickness was reduced to a few micrometers with a diluted solution of HF. Solutions were passed through the OFRR with a syringe pump. These procedures have been described in detail in previous publications [16,22]. A schematic of the experimental setup is shown in Figure 1. The tapered optic fiber with a diameter of approximately 3.5 μm was created by pulling SMF-28 optical fiber under heat. This tapered optical fiber was brought into contact with the OFRR to couple light from a 1,550 nm tunable diode laser from Philips (San Jose, California) into the ring resonator. The laser was scanned at a rate of 0.5 Hz. A photodetector was used to monitor the intensity of light emitted from the end of the tapered fiber cable. When the scanning laser wavelength matches the resonant wavelength, a dip in intensity was observed at the photodetector with a minimum corresponding to the WGM spectral position. This dip in intensity could be used as a sensing signal because the spectral position of the WGM can be tracked in real time. The light intensity measurements were recorded as a voltage using a computer and a data acquisition card from National Instruments (Austin, TX). A LabView interface is used to control the data acquisition rate and laser scanning. The spectral position of the WGM was recorded in real time for post-experiment analysis.

### 3.3. Bulk Refractive Index Sensitivity (BRIS) Characterization of the OFRR

**Figure 6.**
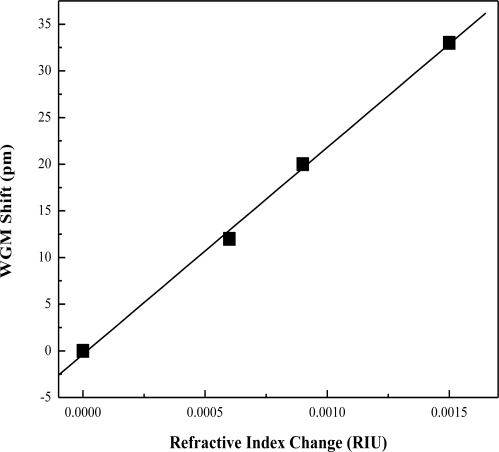
OFRR sensitivity curve with BRIS of 21.9 nm RIU^−1^.

The OFRR utilizes the RI change near the surface of the sensing wall to detect biomolecules. The spectral shift in response to bulk RI changes in the capillary is known as the bulk RI sensitivity (BRIS). In order to establish a relationship between biomolecule buildup and spectral shift, we must establish a bulk sensitivity of the OFRR in units of nm RIU^−1^. First, different concentrations of ethanol in water were run through the OFRR. These solutions had known RI and the resulting shift was recorded for analysis. The increase of RI in these solutions resulted in a larger spectral shift. Fitting the data points with a trend line, we can find that the sensitivity of the OFRR used in our experiments was around 21.9 nm RIU^−1^. Figure 6 shows the characterization curve obtained from the BRIS test.

### 3.4. Functionalization of the OFRR Inner Surface

In order to specifically capture biomolecules, surface functionalization has to be precisely controlled. In this study, antibodies were immobilized on the inner surface of the OFRR to specifically interact with CD4+ and CD8+ receptors located on lymphocytes. First, the surface of the OFRR was charged by passing a very low concentration of HF (2%) followed by ethanol through the inner capillary. Next, a 4% solution of 3-APS in 10% ethanol was passed through the OFRR for 20 minutes to silanize the surface. After rinsing with 10% ethanol, 30 mg of DMP was dissolved in 20 μL of PBS and used to cross link protein G to the surface. Protein G was passed through the OFRR in a 1 mg mL^−1^ of PBS concentration. Protein G serves to orient the antibodies in a way that maximizes the efficiency of receptor interaction. Excess and unbound protein G was rinsed using PBS and then CD4+ or CD8+ antibodies were then pulled through the OFRR.

### 3.5. CD4+ and CD8+ Cell Capture Measurements

After surface functionalization, the OFRR was rinsed with PBS buffer and prepared for CD4+ and CD8+ measurements. White blood cells were isolated from healthy whole blood using a centrifuge. The concentrations of white blood cells were verified using a hemacytometer then adjusted to match CD4+ and CD8+ concentrations of HIV infected individuals. Targeted to the receptors were CD4+ and CD8+ antibodies immobilized in the microfluidic channel of the OFRR. The lymphocyte cells were prepared in PBS buffer at varying concentrations. In each measurement, concentrations of lymphocytes in PBS were passed through the OFRR followed by a PBS rinse to remove unbound cells from the OFRR surface. Anti-CD4 antibody is specific to the CD4+ receptor located on CD4+ Lymphocyte T-helper cells. Anti-CD8 antibody is specific to the CD8+ Cytotoxic T-cells. After each measurement cycle, the OFRR was cleaned with a 2% solution of HF to completely remove all of the functional and biomolecule layers for the surface. After washing, the OFRR can be prepped for the next measurement. The WGM spectral position was recorded and monitored in real time during the buildup of functional layers and receptor measurements. This data was utilized for post-experiment analysis.

## 4. Conclusions

In this work, we have developed label free, rapid, and sensitive optical sensor for use in the whole cell detection of CD4+ and CD8+ T-Lymphocytes, and CD4+ cell lysis detection. We show distinguishable detection ranges around and below 200 CD4+ cells per μL and up to 320 CD4+ cells per μL. We also show a detection range of CD8+ cells from 250 to over 1,000 cells per μL. The concentrations used are at medically significant levels and are comparable with high tier standardized equipment such as flow cytometers. This work could lead to a cost-effective and easy to use device that can be utilized in the field to give an accurate description of HIV progression in resource limited regions.

## References

[1] Sia S.K., Chin C.D., Linder V. (2006). Lab-on-a-chip Devices for Global Health: Past Studies and Future Opportunities. Lab Chip.

[2] Gemignani M., Maiman M., Fruchter R.G., Arrastia C.D., Gibbon D., Ellison T. (1995). CD4 Lymphocytes in Women with Invasive and Preinvasive Cervical Neoplasia. Gynecol. Oncol.

[3] Cheng X., Irimia D., Dixon M., Sekine K., Demirci U., Zamir L., Tompkins R.G., Rodriguez W., Toner M. (2006). A Microfluidic Device for Practical Label-free CD4+ T Cell Counting of HIV-infected Subjects. Lab Chip.

[4] Autran B., Carcelain G., Li T.S., Blanc C., Mathez D., Tubiana R., Katlama C., Debre P., Leibowitch J. (1997). Positive Effects of Combined Antiretroviral Therapy on CD4+ T Cell Homeostasis and Function in Advanced HIV Disease. Science.

[5] Kaufmann D., Pantaleo G., Sudre P., Telenti A. (1998). CD4-Cell Count in HIV-1 Infected Individuals Remaining Viraemic with Highly Active Antiretroviral Therapy (HAART). Lancet.

[6] Gazzard B., Committee B.W. (2006). British HIV Association (BHIVA) Guidelines for the Treatment of HIV-infected Adults with Antiretroviral Therapy. HIV Med.

[7] DHHS Panel on Antiretroviral Guidelines for Adults and Adolescents. Guidelines for the Use of Antiretroviral Agents in HIV-1-infected Adults and Adolescents.

[8] Phillips A.N., Sabin C.A., Elford J., Bofill M., Lee C.A., Janossy G. (1993). CD8 Lymphocyte Counts and Serum Immunoglobulin a Levels Early in HIV Infection as Predictors of CD4 Lymphocyte Depletion During 8 Years of Follow-up. AIDS.

[9] Taylor J.M., Fahey J.L., Detels R., Giorgi J.V. (1989). CD4 Percentage, CD4 Number, and CD4:CD8 Ratio in HIV Infection: Which to Choose and How to Use. J. AIDS.

[10] Rabin R.L., Roederer M., Maldonado Y., Petru A., Herzenberg L.A., Herzenberg L.A. (1995). Altered Representation of Naive and Memory CD8 T Cell Subsets in HIV-infected Children. J. Clin. Invest.

[11] Roederer M., Dubs J.G., Anderson M.T., Raju P.A., Herzenberg L.A., Herzenberg L.A. (1995). CD8 Naive T Cell Counts Decrease Progressively in HIV-infected Adults. J. Clin. Invest.

[12] Livingstone W.J., Moore M., Innes D., Bell J.E., Simmonds P., Study E.H.T. (1996). Frequent Infection of Peripheral Blood CD8-Positive T-Lymphocytes with HIV-1. Lancet.

[13] Strauss K., Hannet I., Engels S., Shiba A., Ward D.M., Ullery S., Jinguji M.G., Valinsky J., Barnett D., Orfao A., Kestens L. (1996). Performance Evaluation of the FACSCount System: A Dedicated System for Clinical Cellular Analysis. Cytometry.

[14] Jokerst J.V., Floriano P.N., Christodoulides N., Simmons G.W., McDevitt J.T. (2008). Integration of Semiconductor Quantum Dots into Nano-bio-chip Systems for Enumeration of CD4+ T Cell Counts as the Point-of-need. Lab Chip.

[15] Wang Y., Kang Y., Xu D., Chon C.H., Barnett L., Kalams S.A., Li D., Li D. (2007). On-Chip Counting the Number and Percentage of CD4+ T-Lymphocytes. Lab Chip.

[16] White I.M., Oveys H., Fan X. (2006). Liquid Core Optical Ring Resonators. Opt. Lett.

[17] Gorodetsky M.L., Illchenko V.S. (1999). Optical Microsphere Resonators: Optimal Coupling to High-Q Whispering-Gallery Modes. J. Opt. Soc. Am. B.

[18] Zhu H., White I.M., Suter J.D., Zourob M., Fan X. (2008). Opto-fluidic Micro-ring Resonator for Sensitive Label-free Viral Detection. Analyst.

[19] Zhu H., White I.M., Suter J.D., Dale P.S., Fan X. (2007). Analysis of Biomolecule Detection with Optofluidic Ring Resonator Sensors. Opt. Express.

[20] Suter J.D., White I.M., Zhu H., Shi H., Caldwell C.W., Fan X. (2007). Label-Free Quantitative DNA Detection Using the Liquid Core Optical Ring Resonator. Biosens. Bioelectron.

[21] Yang G., White I.M., Fan X. (2008). An Opto-fluidic Ring Resonator Biosensor for the Detection of Organophosphorus Pesticides. Sens. Actuator. B Chem.

[22] White I., Gohring J., Fan X. (2007). SERS-Based Detection in an Optofluidic Ring Resonator Platform. Opt. Express.

